# An Exploratory Randomized Clinical Trial to Assess the Effects of Vitamin D and Leucine-Enriched Whey Protein Supplementation on Inflammaging and Gut Eubacterial Biomass in Cardiac Rehabilitation Patients

**DOI:** 10.3390/biom16101484

**Published:** 2026-10-13

**Authors:** Chiara Francesca Gheri, Maria Luisa Eliana Luisi, Laura Lucarini, Barbara Biffi, Sara Madiai, Angela Roccotelli, Renato Zipoli, Silvia Sgambellone, Gustavo Provensi, Claudio Macchi, Emanuela Masini, Maria Teresa Ceccherini

**Affiliations:** 1 Dietetic and Clinical Nutrition Service, IRCCS Fondazione Don Carlo Gnocchi ETS, Via di Scandicci 269, 50143 Florence, Italy; cgheri@dongnocchi.it (C.F.G.); mluisi@dongnocchi.it (M.L.E.L.); bbiffi@dongnocchi.it (B.B.); smadiai@dongnocchi.it (S.M.); 2 Department of Neurofarba, Section of Pharmacology and Toxicology, University of Florence, Viale Pieraccini 6, 50139 Florence, Italy; silvia.sgambellone@unifi.it (S.S.); gustavo.provensi@unifi.it (G.P.); emanuela.masini@unifi.it (E.M.); 3 Department of Life Sciences, Health and Health Professions, Link Campus University, 00165 Rome, Italy; 4 Department of Agriculture, Food, Environment and Forestry (DAGRI), University of Florence, Piazzale delle Cascine 18, 50144 Florence, Italy; angela.roccotelli@unifi.it (A.R.); mariateresa.ceccherini@unifi.it (M.T.C.); 5 Cardiac Rehabilitation Unit, IRCCS Fondazione Don Carlo Gnocchi ETS, Via di Scandicci 269, 50143 Florence, Italy; rzipoli@dongnocchi.it; 6 Department of Rehabilitation, IRCCS Fondazione Don Carlo Gnocchi ETS, Via di Scandicci 269, 50143 Florence, Italy; cmacchi@dongnocchi.it

**Keywords:** whey protein, rehabilitation, IL-6, CRP, microbiota, inflammaging, cardiovascular disease, non-communicable disease

## Abstract

Whey protein (WP) supplementation could affect low-grade inflammation (inflammaging) and gut microbiome dysbiosis, both of which play key roles in non-communicable diseases (NCDs). Twenty-seven cardiac rehabilitation patients were randomly allocated to two groups: the case group (CA) (n = 14) received 80 g/die vitamin D and leucine-enriched WP supplement, combined with a standard hospital diet, while the control group (CO, n = 13) received the standard hospital diet alone. Blood and stool samples were collected at baseline (T0) and after two weeks (T1). At T1, serum levels of interleukin 6 (IL-6) and C-reactive protein (CRP) significantly decreased in the CA group. Ferritin levels significantly decreased within both groups and between the two groups. No significant differences were observed for IL-1β, tumor necrosis factor-α (TNF-α), or IL-10. A clear difference in the bacterial intestinal microbiome composition between the case group at T1 (CAT1) and at T0 (CAT0), and control group at T0 (COT0) and at T1 (COT1), was observed. The eubacterial *rpo*B copy number, which was quantified by qPCR, significantly increased between CAT0 and CAT1, in contrast to the *Lactobacillus* spp. *rpo*B copy number. Our exploratory results showed that vitamin D and leucine-enriched WP supplementation could have a potential effect on chronic inflammation and gut microbiota composition in cardiac rehabilitation patients.

## 1. Introduction

In Western countries, life spans are high; most older people develop a low-grade pro-inflammatory status termed “inflammaging”, characterized by elevated circulating levels of pro-inflammatory markers [1].

A sustained, low-grade inflammatory state is a key contributor to the onset and progression of non-communicable diseases (NCDs), as shown by previous studies reporting positive associations between high serum levels of inflammatory markers, such as IL-6 and TNF-α, low-grade inflammation, and NCDs [2].

Inflammaging is recognized as both a risk factor and a pathogenic mechanism for the onset of cardiovascular disease (CVD) [1]; it is associated with an increased risk of morbidity and mortality [2]. As a leading cause of death and disability globally, CVD represents a major burden on national healthcare systems. Numerous environmental factors influence chronic, low-grade, inflammatory states, with an estimated 40% of CVD cases attributable to unhealthy lifestyle choices and dietary habits [3]. Consequently, diet is recognized as a critical modifiable risk factor for cardiovascular health. Healthy dietary patterns, such as the Mediterranean diet, are characterized by high intake of fibers, vitamins, lean proteins, low glycemic carbohydrates, antioxidants, and mono- and polyunsaturated fatty acids, and have been shown to decrease inflammatory markers and overall CVD risk [3,4]. Dairy products represent a key component of the Mediterranean diet. Epidemiological evidence indicates that dairy consumption exerts a beneficial impact on cardiometabolic health, with no observed adverse effects [5]. Milk is considered a functional food with many different properties, such as antimicrobial, immunological and antitumorigenic properties, and its bioactive components have been studied as potential prebiotics [6]. Among these bioactive compounds, whey proteins (WP) account for about 20% of the total high-quality protein in bovine milk. WP exhibits both primary and secondary bioactivities upon digestion and has been evaluated as a potential dietary modulator of chronic low-grade inflammation.

WP are also rich in branched-chain amino acids such as leucine. Recent clinical trials and systematic reviews underscore the efficacy of targeted amino acid and micronutrient enrichment in cardiovascular populations. For instance, Kawakubo et al. demonstrated that leucine-focused nutritional guidance significantly improves functional recovery in cardiac rehabilitation [7]. Furthermore, a systematic review by Pellegrini et al. confirmed that protein supplementation positively modulates body composition and inflammatory tone in cardiovascular disease patients [8], reinforcing earlier findings by Lieberman et al. regarding whey protein and vitamin D synergy in geriatric sarcopenia [9]. Nutritional recommendations for adults over 65 years suggest 25–30 g of high-quality protein, containing 2.8–3 g of leucine, at least twice a day [10]. In sarcopenic older adults, particularly those unable to exercise, oral nutritional supplementation with vitamin D and leucine-enriched WP improved muscle mass and physical function [11]. A recent systematic review and meta-analysis showed that WP combined with vitamin D improved lean mass, muscle strength, and physical performance, particularly in short-term interventions (<12 weeks) and in individuals not engaged in resistance exercise, across both sarcopenic/frail and healthy older populations [12]. In contrast, WP supplementation alone had no effect on lean mass or muscle strength in healthy older adults, providing effects only for improving lean mass and physical function in sarcopenic/frail older subjects [12].

Regarding the anti-inflammatory effect of WP supplementation, substantial heterogeneity exists across studies. Findings suggest that these anti-inflammatory properties depend on patients’ characteristics, daily WP dosage, protein form (hydrolyzed or intact WP) and treatment duration. For example, older patients with acute ischemic stroke who received hydrolyzed WP for five days showed decreased serum IL-6 levels and increased glutathione levels [13]. Similarly, a systematic review reported a significant reduction in serum CRP levels following intact WP supplementation, but only when administered at high doses or in individuals with elevated CRP levels [14]. Moreover, a recent meta-analysis demonstrated a significant reduction in serum IL-6 levels following intact WP supplementation in older adults [15].

Unhealthy dietary habits and drugs are major causes of gut microbiota dysbiosis, which in turn plays a key role in inflammaging and in the pathogenesis of numerous non-communicable diseases (NCDs) [16].

In recent years, the gut microbiota in older adults has been shown to regulate chronic inflammation by acting as a guardian of the intestinal barrier and a producer of immunomodulatory metabolites such as short-chain fatty acids (SCFAs). Dysbiosis disrupts this balance, turning the gut into a primary source of pro-inflammatory factors that translocate into the systemic circulation, directly contributing to the development of sarcopenia, neuroinflammation, and frailty in older adults [17,18].

Whey proteins (WP) possess antimicrobial, immunomodulatory, and prebiotic properties that can help to restore microbial balance [6]. Recent research indicates that, in addition to modulating microbiome, WP play a significant role in strengthening intestinal barrier integrity and regulating host metabolism. Evidence from in vitro, in vivo, and clinical studies demonstrate that WP can promote the growth of beneficial bacteria, such as *Bifidobacterium*, *Lactobacillus*, while simultaneously inhibiting harmful ones [19,20]. Furthermore, WP supplementation has been shown to alleviate conditions associated with dysbiosis—such as colitis, obesity, and allergies—by enhancing microbial diversity, increasing short-chain fatty acid production, strengthening the mucosal barrier, and modulating immune responses, from infancy through adulthood. However, these effects vary, depending on the WP composition and other characteristics such as the individual’s microbiota [19]. Therefore, further research is needed to confirm the benefits of WP and optimize their clinical application [6,19,20,21,22].

The present study aimed to evaluate the effect of high-dose daily WP supplementation on the inflammatory marker IL-6 in cardiac rehabilitation patients. Secondary goals were to assess whether WP supplementation modulates the following inflammatory markers: (i) the pro-inflammatory cytokine interleukin 1β (IL-1β); (ii) tumor necrosis factor α (TNF-α); (iii) C reactive protein (CRP); (iv) ferritin; and (v) anti-inflammatory interleukin 10 (IL-10). Furthermore, we investigated the effect of WP supplementation on the gut bacteriome, with a focus on lactic acid bacteria (LAB), as these are recognized as probiotic agents with an important role in the animal gut ecosystem and have been shown to be anti-inflammatory taxa, associated with reduced inflammaging and greater physical resilience [23,24]. These bacteria are key to maintaining intestinal health in humans, acting as beneficial bacteria capable of modulating the metabolism and barrier function of the intestine. Indeed, the stimulation of *Lactobacillus* (in synergy with *Bifidobacterium*) promotes the production of short-chain fatty acids (SCFAs) that promote the health and integrity of the intestinal mucosa, both in normal-weight and obese subjects [25]. In particular, the growth and presence of *Lactobacillus* in the intestine are supported by the intake of whey proteins (WP), which exert a prebiotic action by selecting beneficial bacterial flora to the detriment of potentially harmful or pro-inflammatory bacteria [19,26]. These aspects influenced our decision to monitor the *Lactobacillus* population, albeit in an exploratory manner.

## 2. Materials and Methods

### 2.1. Patient Enrolment

An open-label, non-blinded, non-commercial, multi-center, randomized, controlled trial was conducted. Patients were recruited at the Cardiac Rehabilitation Unit of the IRCCS (Istituto di Ricovero e Cura a Carattere Scientifico–Scientific Institute for Research, Hospitalization and Care) Fondazione Don Carlo Gnocchi (FDG) ETS (Ente del Terzo Settore–Third Sector Entity) of Florence from May 2018 to December 2019.

The Dietetic and Clinical Nutrition Service (DCNS), composed of physicians and dietitians, operates within the IRCCS Fondazione Don Carlo Gnocchi of Florence, a general rehabilitation and research hospital that admits patients primarily referred by acute care hospitals. The institution provides rehabilitation programs with direct public healthcare coverage.

The Ethics Committee of the IRCCS Fondazione Don Carlo Gnocchi approved the study (approval number 13_4/4/2018), which was performed in accordance with the principles of the Helsinki Declaration. The number of the ClinicalTrials.gov Identifier is NCT04738994.

Each patient was informed of the purpose of the study and provided written informed consent.

Inclusion criteria were: admission for cardiac rehabilitation; signed informed consent; and female and male patients aged ≥50 years.

Exclusion criteria were: inability to eat orally; eating disorders; levodopa therapy; gastrointestinal malignant diseases; severe kidney and liver failure; recent (1 month or ongoing) antibiotic therapy; and refusal to participate.

Patients were divided into two groups: the control group (CO) received a standard hospital diet according to the specific patient’s clinical needs; and the case group (CA) received a standard hospital diet according to the specific patient’s clinical needs plus 80 g/die (two servings/die) of whey proteins, leucine, and vitamin D-enriched oral nutritional supplement dissolved in 125 mL of water [10].

The low glycemic index (≤55) whey protein-based food for special medical purposes provided, per serving (40 g of powder): 150 kcal (1 Kcal/ml); 20 g of 100% whey protein isolate; 2.8 g of leucine; 20 µg of vitamin D3; and 3 g of fat, minerals, vitamins and fibers.

Patients took the WP, leucine and vitamin D-enriched oral nutritional supplements between meals, twice a day, in the morning and in the afternoon.

A composite formula (WP + leucine + vitamin D + dietary fiber) was selected because sarcopenia and inflammaging in older cardiac patients involve multifactorial signaling defects [10]. Whey protein provides essential amino acids; leucine directly activates the mTORC1 muscle protein synthesis pathway [27]; vitamin D modulates immune cell receptor expression [28]; and dietary fiber serves as a substrate for short-chain fatty acid (SCFA)-producing gut microbes [29].

Patients received a nutritional plan tailored to their underlying disease, current clinical needs, and on-going treatments, in alignment with prior recommendations from the referring hospital and the institutional dietary protocols developed by the DCNS, which encompass unrestricted or therapeutic diets, as previously described in greater detail elsewhere [30]. Nutritional care plans were adapted during hospitalization in response to changes in the patient’s clinical needs. Additionally, control (CO) group patients with hypovitaminosis D received weekly oral drops of vitamin D3.

According to a standardized institutional protocol, all patients participated in a comprehensive multidisciplinary rehabilitation program during the hospitalization, which comprised a tailored exercise-based physiotherapy program consisting of two one-hour sessions per day, 5 days aweek. According to the patient’s specific rehabilitation goals and clinical needs, exercises varied in intensity and difficulty and were conducted under a therapist’s supervision [31].

In an attempt to minimize bias, participants were randomly assigned to either the case or control group, thereby ensuring that the two groups were as comparable as possible with respect to patient characteristics that may have an impact on outcomes. All patients and investigators were aware of the group assignment.

In the CA group, WP supplementation was started as soon as possible after enrolment and randomization.

At enrolment (T0) all patients received the same procedures and evaluations.

At T0, all patients received anamnestic and anthropometric assessment, and blood and stool samples were collected.

Body height, expressed in meters, was measured using a wall-mounted stadiometer (SECA, Hamburg, Germany) with patients standing barefoot with their heads positioned in the Frankfurt-horizontal plane. Body weight, expressed in kg, was measured on a calibrated platform scale (capacity 150 kg, graduation 0.1 kg, SECA, Hamburg, Germany), with patients wearing light clothes.

Body Mass Index (BMI) was calculated as [weight (kg)]/[height (m)]^2^.

Venous blood was collected in the morning after an overnight fast through an indwelling catheter inserted in the antecubital vein. Laboratory data included fasting glucose; total-, LDL and HDL cholesterol; triglycerides; creatinine; C reactive protein (CRP); iron; transferrin; ferritin; 25-hydroxy-vitamin D [25(OH)D]; pro-inflammatory cytokines IL-6, IL-1β, TNF-α, and anti-inflammatory cytokine IL-10.

All the same evaluations, except for the height measurement, were repeated after two weeks (T1). The time frame upon which to assess the measurements was two weeks because, as a rule, this is the hospitalization length required to attend the cardiac rehabilitation program in our hospital.

### 2.2. Biochemical Analysis

The determination of fasting glucose, total, LDL, and HDL cholesterol, triglycerides, creatinine, C reactive protein (CRP), iron, transferrin, ferritin, and 25-hydroxy-vitamin D [25(OH)D] was performed by Synlab Toscana, Italy, the reference laboratory of our Centre.

#### 2.2.1. Determination of Cytokine Concentrations in Human Plasma with Bead-Based Multiplex Immunoassays

The quantitative determination of the IL-1β, IL-6, IL-10, and TNFα cytokine’s concentrations was performed by a bead-based multiplex immunoassay (Milliplex MAP KIT, Cat.N. HCYTA-60k, Millipore, MA, USA) using a Bioplex 200 system (Biorad Laboratories, Hercules, CA, USA) [32]. In brief, human plasma was added to antibody-conjugated beads directed against the cytokines listed above in a 96-well filter plate. After an overnight incubation at 2 °C, the plate was washed, and biotinylated anti-cytokine antibody solution was added before 1 h incubation. The plate was then washed and streptavidin-conjugated PE was added. After a final wash, each well was suspended with assay buffer and analyzed with the Bioplex 200 system. Standard curves were derived from various concentrations of the different cytokine standards and followed the same protocol as the plasma samples. The concentration of each cytokine (pg/mL) in each plate well was calculated using Bioplex200 System Array (Bio-Rad, Hercules, CA. USA). 

Not all samples were analyzed for all tests, due to insufficient plasma in some samples.

#### 2.2.2. Gut Microbiome DNA Extraction, Quantification, and 16S rDNA Sequencing

Total DNA [33] was extracted from fecal samples by following the QIAamp DNA Stool Mini Kit instructions (Qiagen, Hilden, Germany) and was quantified with a Qubit^®^ 2.0 fluorometer (Invitrogen, Carlsbad, CA, USA). The yield was calculated as μg DNAg^−1^ feces. Quantitative PCR (qPCR) was conducted using the eubacterial primers *rpo*B1698f and *rpo*B2041r and the specific LAB primers *rpo*B1, *rpo*B1o, and *rpo*B2, for the RNA-polymerase β subunit encoding gene, present in a single copy per genome [34,35], on 40 ng DNA for all the samples; the negative control contained only ddH_2_O. Reactions were performed in a CFX Connect 96 apparatus (Bio-Rad) and the results were analyzed by the manufacturer’s software. Amplification was carried out in a 25 μL final volume containing 7.5 μM of each primer, 2.5 μg of BSA, 1X iTaq™ Universal SYBR^®^ Green Supermix, and sterile ddH0_2_O to reach the appropriate volume. Amplification was performed in 96-well micro titer plates (Bio-Rad). The cycle was 95 °C 3 min followed by 35 cycles of 95 °C 1 min, 45 °C 1 min, and 72 °C 1 min. A melting curve program was then run, in which measurements were made at 0.5 °C temperature increments every 10 s within a range of 60–100 °C. A *rpo*B amplified and purified fragment (from a mix of LABs of a commercial probiotic formulation—Lactoflorene^®^ Plus and Montefarmaco OTC—was used as the standard [36]. The standard curve was developed by plotting the logarithm of known concentrations (tenfold dilution series in triplicate from 6.5 × 10^−1^ to 6.5 × 10^−5^ in 25 μL reaction) of the *rpo*B fragment against the threshold cycle (Ct) values [37]. The mean was assessed across all cases and controls as *rpo*B copies ng^−1^ DNA, at two time intervals, T0 (enrollment) and T1 (after two weeks).

Sample data were reported as the average of 3 replicas. Differences in Eubacteria and *Lactobacillus* sequence amounts between T0 and T1 were assessed in both Case (CA) and Control (CO) groups. The significance of the differences observed was calculated using the *t*-test in PAST4.

NGS sequencing and library preparation were performed by Novogene Company (Novogene, Beijing, China) in the 16S rDNA V3-V4 region, on the NovaSeq PE 250 System.

### 2.3. Bioinformatic Analysis

Sequencing data processing paired-end reads were assigned to samples based on their unique barcodes, truncated by cutting off the barcode and primer sequences, and merged using FLASH (V1.2.7). Quality filtering on the raw tags was performed to obtain high-quality clean tags according to the quality-controlled process Qiime (V1.7.0). The tags were compared with the reference database (Gold database) using the UCHIME algorithm (UCHIME Algorithm) to detect chimera sequences; then, the chimera sequences were removed, obtaining, finally, the effective tags.

OTU cluster and taxonomic annotation sequence analyses were performed by Uparse software (Uparse, v7.0.1001), using all the effective tags. Sequences with ≥97% similarity were assigned to the same OTUs. A representative sequence for each OTU was screened for further annotation. For each representative sequence, Mothur software (v.1.43.0) was performed against the SSU rRNA database of the SILVA Database for species annotation at each taxonomic rank. To obtain the phylogenetic relationship of all OTU representative sequences, MUSCLE alignment software (Version 3.8.31) was used to compare multiple sequences rapidly. OTU abundance information was normalized using a standard sequence number corresponding to the sample with the fewest sequences. We then selected the top 10 taxa at the Phylum and Genus taxonomic rank for each sample to obtain the relative abundance of taxa [38].

### 2.4. Statistical Analysis

Sample size calculation was performed to assess the minimum number of participants to be included in the study and was based on previous study of De Aguilar-Nascimiento et al. [13]. IL-6 serum levels at T0 and T 1 were the primary outcome, in accordance with previous evidence [13,39,40].

Sample size was calculated considering that 80% was the probability that the study revealed as a difference in serum IL-6 levels between the two groups (CO and CA at T0), with a significance level of 0.05, assuming that the set power was 0.84. The minimum sample size for this study was 16 patients.

Data shown in figures are expressed as individual points for each individual (aligned plot). The bar graphs represent the mean ± standard error of the mean (S.E.M). Experimental data were also used to determine the variation in T1–T0, which was calculated by subtracting the values measured at baseline (T0) from the corresponding measurement determined at the final time-point (T1).

Two-way repeated measures ANOVA with time-points (T0 vs. T1) and condition (cases vs. controls) as factors, followed by Bonferroni’s multiple comparison test, were applied to assess differences in individual determinations, while the unpaired Student’s *t*-test was used to evaluate differences in the variation data. *p* values lower than 0.05 were considered statistically significant. Statistical analysis was performed using GraphPad Prism(R) software (version 9.3.1).

For an exploratory analysis of the bacteriome and lactobacilli, the same parameters were adequately powered for microbiome analyses.

## 3. Results

A total of twenty-seven patients admitted to the Cardiac Rehabilitation Unit of the IRCCS Fondazione Don Carlo Gnocchi of Florence were enrolled from May 2018 to December 2019.

The mean age of all enrolled patients was 70.7 ± 8 (range 55–84) years. The demographic and clinical characteristics of patients are described in Table 1. While the upper age limit differed slightly between groups (84 vs. 77 years), the mean overall age did not show a statistically significant difference between the intervention (65.4 ± 7.2 years) and control (67.1 ± 8.1 years) groups (*p* > 0.05). To ensure that this age range spread did not bias our findings, statistical comparisons (ΔT1–T0) were evaluated.

Medications were similar in both groups at baseline and at discharge.

### 3.1. Anthropometric and Hematochemical Parameters

**Figure 1:**
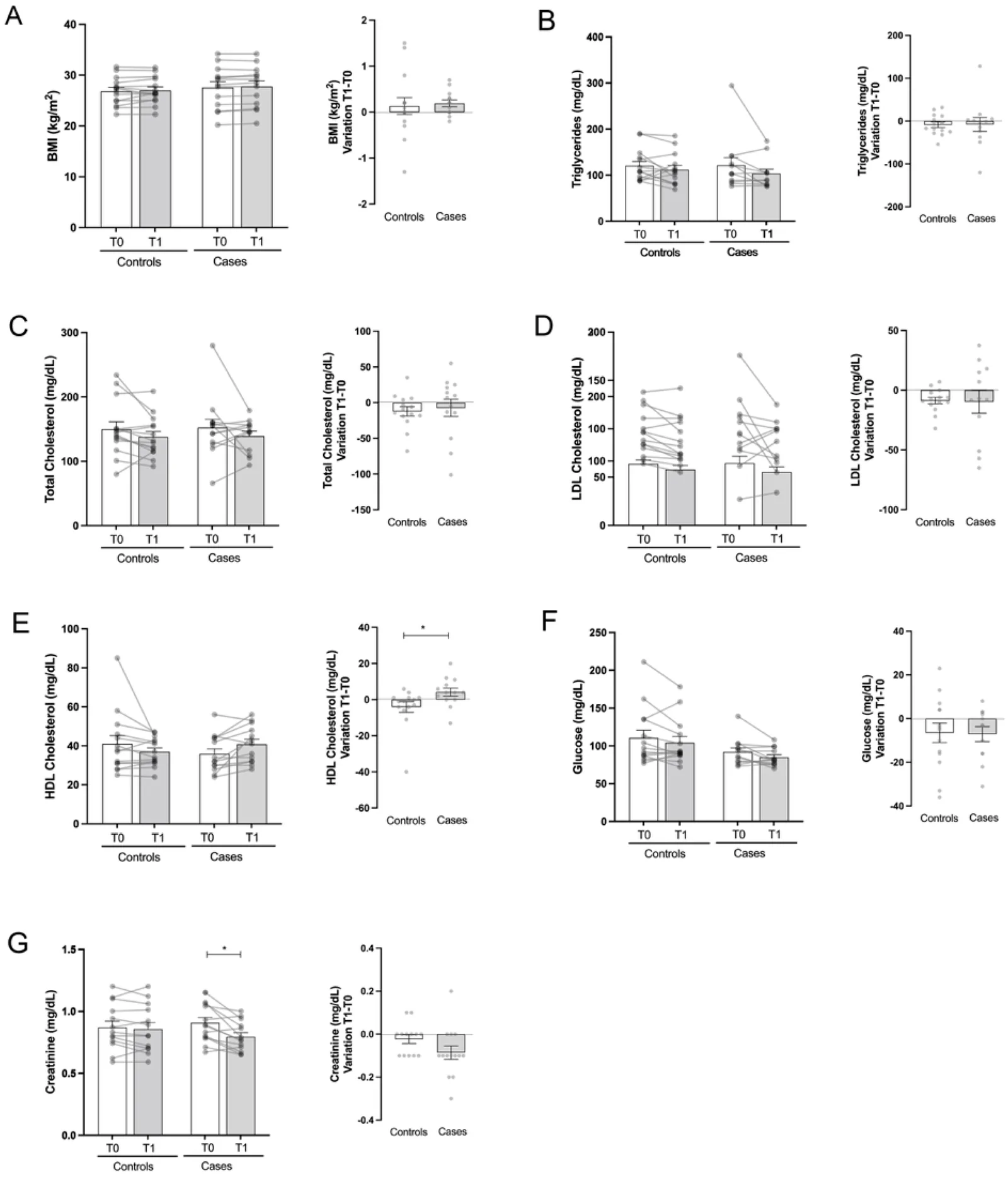
Anthropometric and hematochemical parameters. Analysis of BMI (**A**); Triglycerides (**B**); Total Cholesterol (**C**); LDL Cholesterol (**D**); HDL Cholesterol (**E**); Glucose (**F**) and Creatinine (**G**) content in plasma samples of controls and cases at T0 and T1. Variation T1–T0 values are calculated by subtracting the final value after 2 weeks from the corresponding initial value at baseline. Data are mean ± S.E.M. * *p* < 0.05. Two-way repeated measures ANOVA followed by Bonferroni’s post hoc test were used for the analysis of differences among the groups; *t*-test was used for the variation analysis (ΔT1–T0).

In both control and case groups, body mass index (BMI), total cholesterol, low density lipoprotein (LDL) cholesterol, and triglycerides were virtually unchanged from baseline (TO) to two weeks (T1) (Figure 1).

Conversely, high-density lipoprotein (HDL) cholesterol exhibited an upward trend from T0 to T1 in case group, whereas it demonstrated a downward trend in the control group.

The change over time (ΔT1–T0) differed significantly between the two groups.

Fasting glucose was almost identical at T1 when compared to T0 in both controls and cases.

Finally, serum creatinine levels in controls had a decreasing trend and, in cases, were significantly lower from T0 to T1 (Figure 1).

### 3.2. Inflammatory Markers

**Figure 2:**
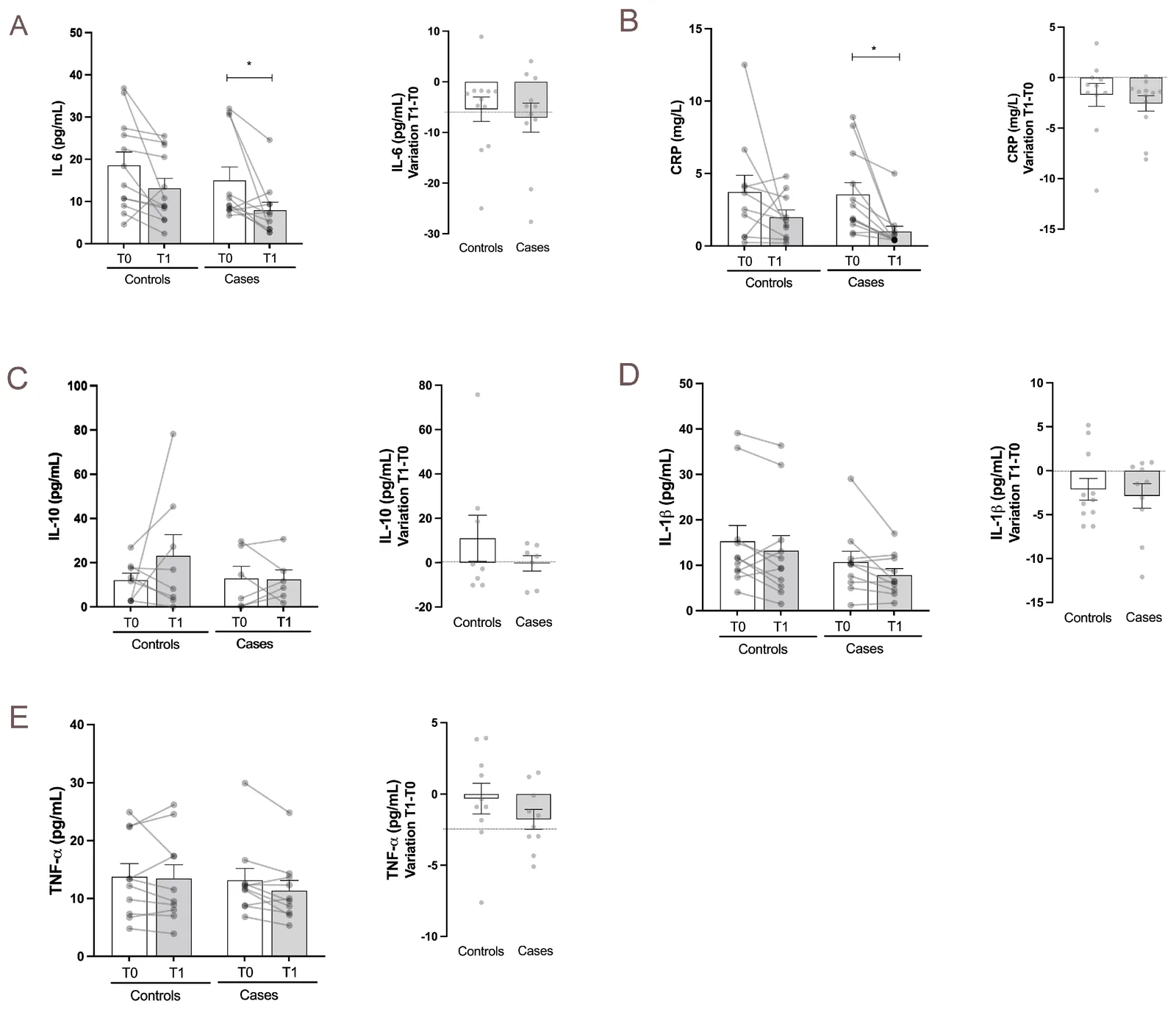
Determination of inflammatory markers: Analysis of IL-6 (**A**); CRP (**B**); IL-10 (**C**); IL-1β (**D**); and TNF-α (**E**) content in plasma samples of controls and cases at T0 and T1. Variation T1–T0 values are calculated by subtracting the final value after 2 weeks from the corresponding initial value at baseline. Data are mean ± S.E.M. * *p* < 0.05. Two-way repeated measures ANOVA followed by Bonferroni’s post hoc test were used for the analysis of differences among the groups; *t*-test was used for the variation analysis (ΔT1–T0).

IL-6 and CRP were significantly lower in cases at T1 when compared to T0; however, we failed to observe any significant difference between the two groups (Figure 2).

Mean IL-1β levels of both groups showed a decreasing trend from T0 to T1, although these differences were not significant. Levels of TNF-α remained almost identical in controls and showed a decreasing trend in cases from T0 to T1. Anti-inflammatory cytokine IL-10 was increased in the controls and almost the same in cases from T0 to T1.

### 3.3. Iron Status

The WP supplement administered in the study was also enriched with iron.

**Figure 3:**
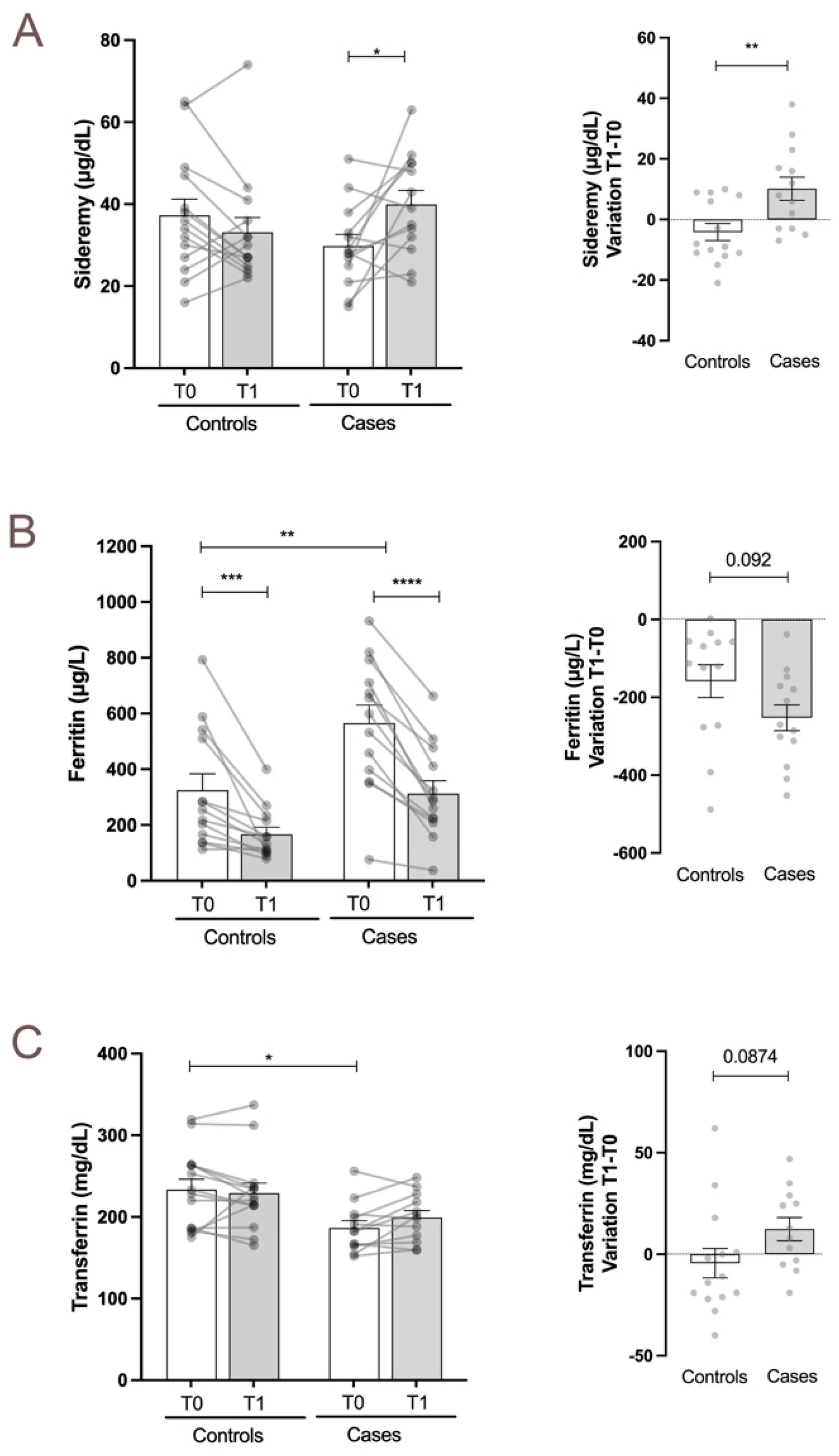
Iron status. Analysis of Sideremy (**A**); Ferritin (**B**) and Transferrin (**C**) content in plasma samples of controls and cases at T0 and T1. Variation T1–T0 values are calculated by subtracting the final value after 2 weeks from the corresponding initial value at baseline. Data are mean ± S.E.M. * *p* < 0.05, ** *p* < 0.01, *** *p* < 0.001 and **** *p* < 0.0001. Two-way repeated measures ANOVA followed by Bonferroni’s post hoc test were used for the analysis of differences among the groups; *t*-test was used for the variation analysis (ΔT1–T0).

In the case group, serum iron levels increased significantly from T0 to T1, remaining within the reference laboratory ranges (Figure 3).

Conversely, the control group exhibited a decreasing trend in iron levels from T0 to T1, yielding a significant difference in change over time (ΔT1–T0) between the two groups.

Transferrin levels mirrored changes in serum iron while remaining within normal reference limits. Specifically, transferrin showed a decreasing trend from T0 to T1 in the control group, compared to an increasing trend observed in the case group. The difference in change (ΔT1–T0) showed a significant difference between the two groups.

Finally, at T1, acute phase reactant protein ferritin was significantly decreased in both groups; the difference was also significant between the two groups.

### 3.4. Vitamin D Status

**Figure 4:**
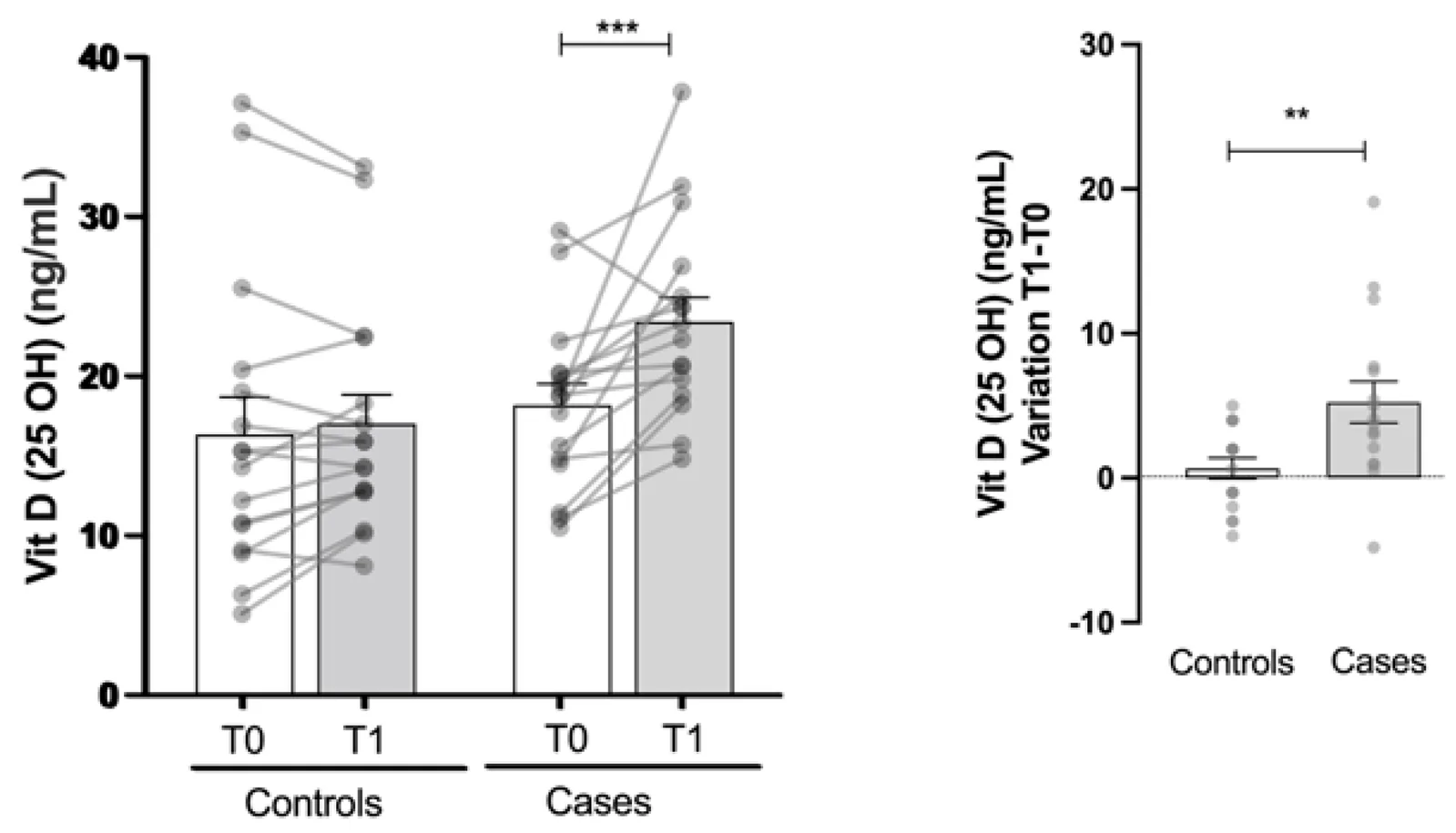
Vitamin D status. Data are mean ± S.E.M. Variation T1–T0 values are calculated by subtracting the final value after 2 weeks from the corresponding initial value at baseline. ** *p* < 0.01 and *** *p* < 0.001. Two-way repeated measures ANOVA followed by Bonferroni’s post hoc test were used for the analysis of differences among the groups; *t*-test was used for the variation analysis (ΔT1–T0).

Vitamin D increased in both groups from T0 to T1 and, in cases, increased significantly (Figure 4). The ΔT1–T0 showed a significant difference between the two groups.

### 3.5. Gut Microbiome Analysis

After a rigorous quality check and preprocessing, which removed about 20% of the sequences, all sequences were normalized to 7000 sequences per sample, resulting in a total of 187,807 OTUs, with a median of 1970 OTUs per sample.

#### 3.5.1. The Relative Abundance of Gut Bacteriome

To investigate the effect of the WP supplement on gut bacteriome composition, we characterized the fecal bacterial community at family and genus taxonomic levels of the four groups of individuals over two weeks.

**Figure 5:**
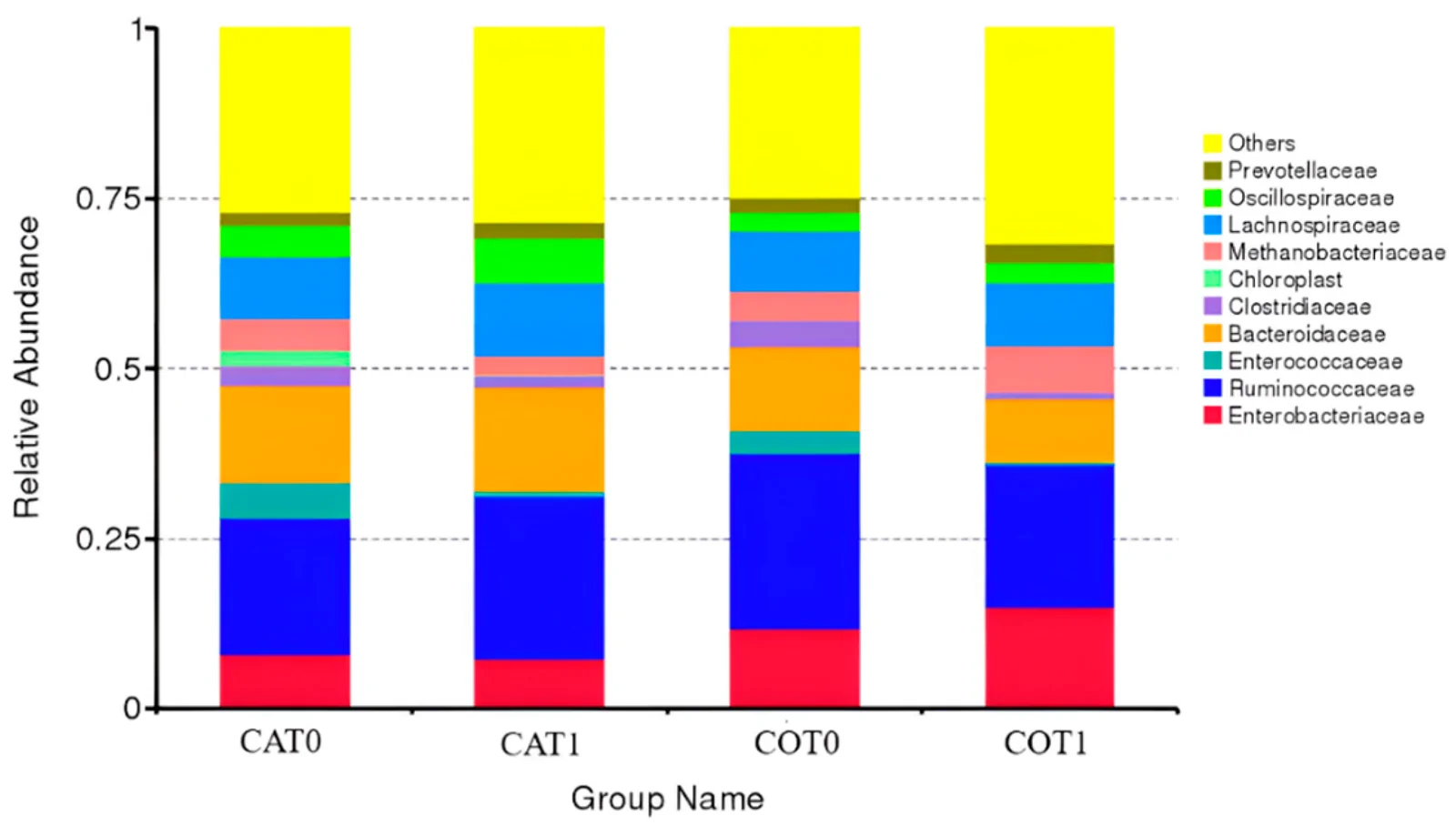
Family-level abundance. Abbreviations: COT0, COT1: control non-treated patients at time 0 (0 days) and time 1 (after two weeks) from time of entrance to IRCCS Fondazione Don Carlo Gnocchi. CAT0, CAT1: WP-treated patients at time 0 and 1 as described above.

Figure 5 shows the relative abundances at family level. The principal 10 families, in order of relative abundance, included: *Enterobacteriaceae*, *Ruminococcaceae*, *Enterococcaceae, Bacteroidaceae*, *Clostridiaceae*, *Chloropast*, *Methanobacteriaceae*, *Lachnospiraceae*, *Oscillospiraceae*, and *Prevotellaceae*. The three most abundant were *Ruminococcaceae*, *Enterobacteriaceae,* and *Bacteroidaceae*.

**Figure 6:**
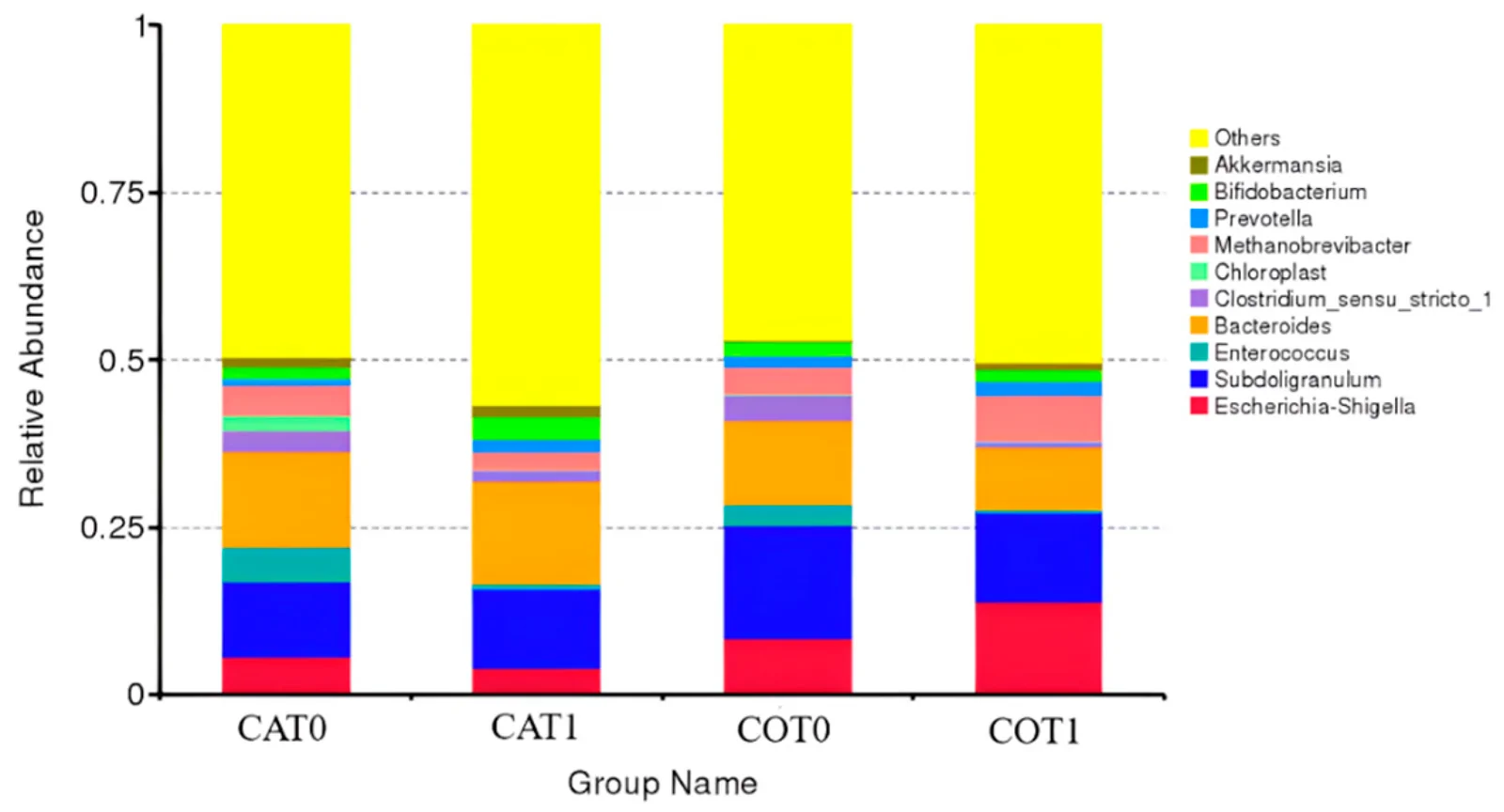
Genus-level abundance. Abbreviations: COT0, COT1: control non-treated patients at time 0 (0 days) and time 1 (after two weeks) from time of entrance to IRCCS Fondazione Don Carlo Gnocchi. CAT0, CAT1: WP-treated patients at time 0 and 1, as described above.

The three most abundant genera, within the most represented ten, were *Subdoligranulum* (belonging to the *Ruminococcaceae* family), *Bacteroides*, and *Escherichia-Shigella* (Figure 6).

**Figure 7:**
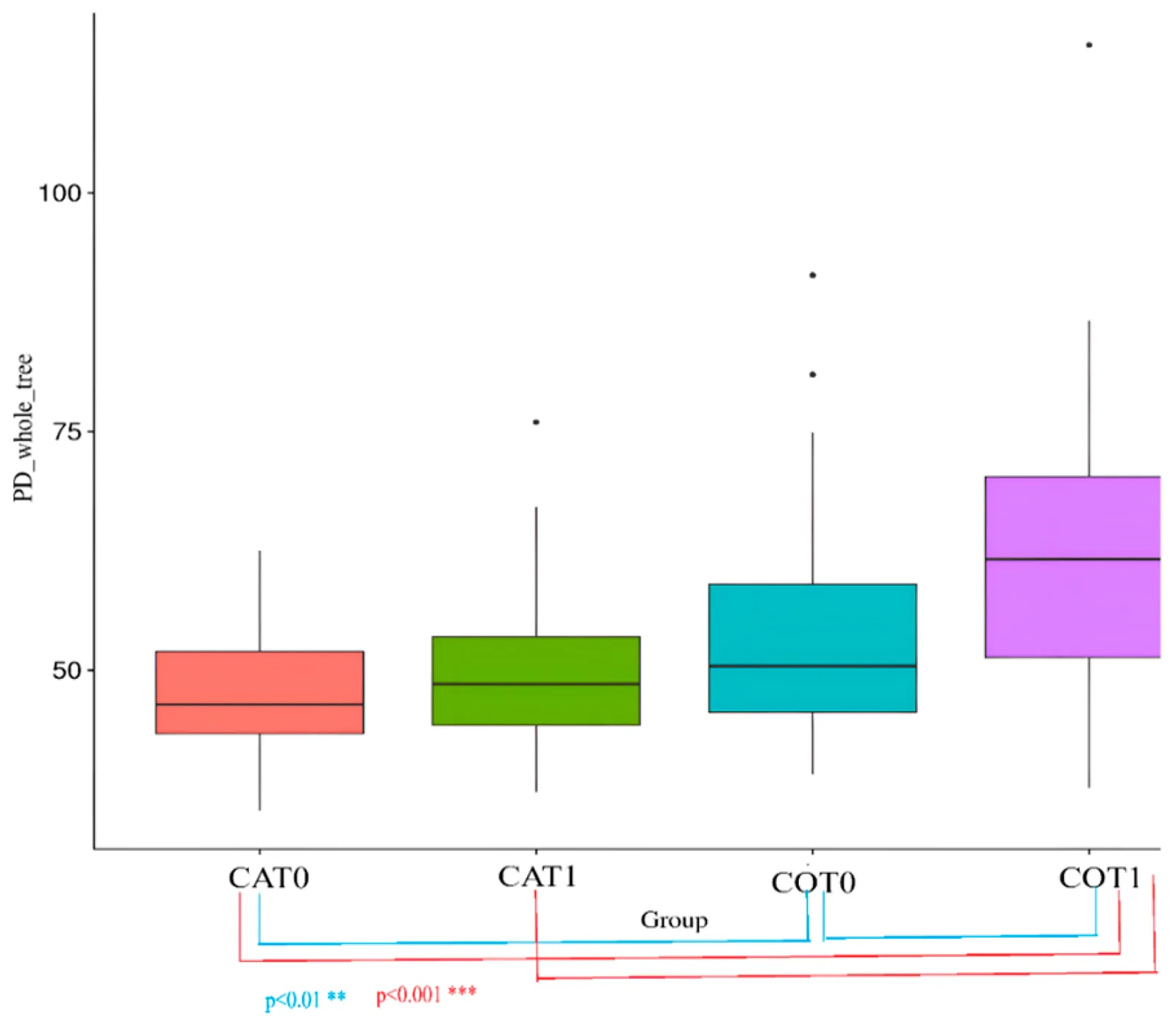
Alpha diversity analysis. Abbreviations: COT0, COT1: control non-treated patients at time 0 (0 days) and time 1 (after two weeks) from time of entrance to IRCCS Fondazione Don Carlo Gnocchi. CAT0, CAT1: WP-treated patients at time 0 and 1 as described above.

The alpha diversity analysis, calculated as phylogenetic diversity whole tree (PD), showed that (Figure 7):

CAT0–CAT1 were not significantly different. They evidenced a similar richness of OTUs, that is, a similar bacterial composition;

COT0–COT1: there was an increase in alpha diversity in COT1, and composition was significantly different (*p* < 0.01);

CAT0–COT0: increase in alpha diversity in COT0 and significantly different (*p* < 0.01);

CAT0–COT1: increase in diversity in COT1 and significantly different (*p* < 0.001);

CAT1–COT1: increase in diversity in COT1 and significantly different (*p* < 0.001).

**Figure 8:**
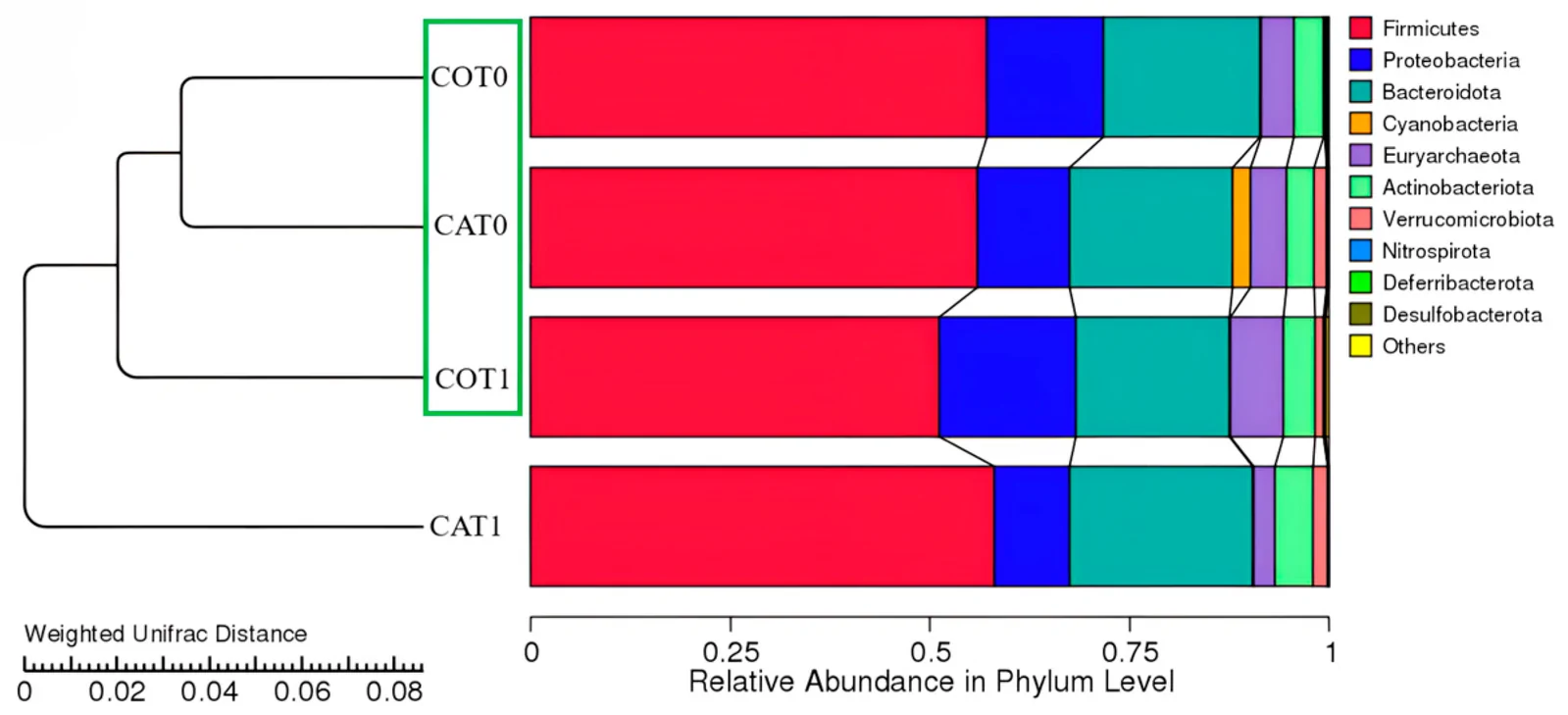
UPGMA tree based on weighted UniFrac distance at the phylum level. On the left is the UPGMA cluster tree structure; on the right is the species relative abundance distribution at the phylum level for each sample. The phylogenetic relationship of the bacterial isolates was determined by PCR sequencing portions of the 16S rRNA gene. Abbreviations: COT0, COT1: control non-treated patients at time 0 (0 days) and time 1 (after two weeks) from time of entrance to IRCCS Fondazione Don Carlo Gnocchi. CAT0, CAT1: WP-treated patients at time 0 and 1 as described above.

The beta diversity, representing the diversity between groups of the bacterial community, was evaluated by the unweighted pair-group method with arithmetic mean (UPGMA), which was used to solve the classification [43]. Here, the UPGMA tree, based on weighted UniFrac distance at the phylum level (Figure 8), showed that the bacterial communities were divided into two major groups: COT0, CAT0 and COT1, where COT1 deviated from COT0 more than CAT0; and CAT1, uniquely treated with WP, was distanced phylogenetically from the untreated groups.

**Figure 9:**
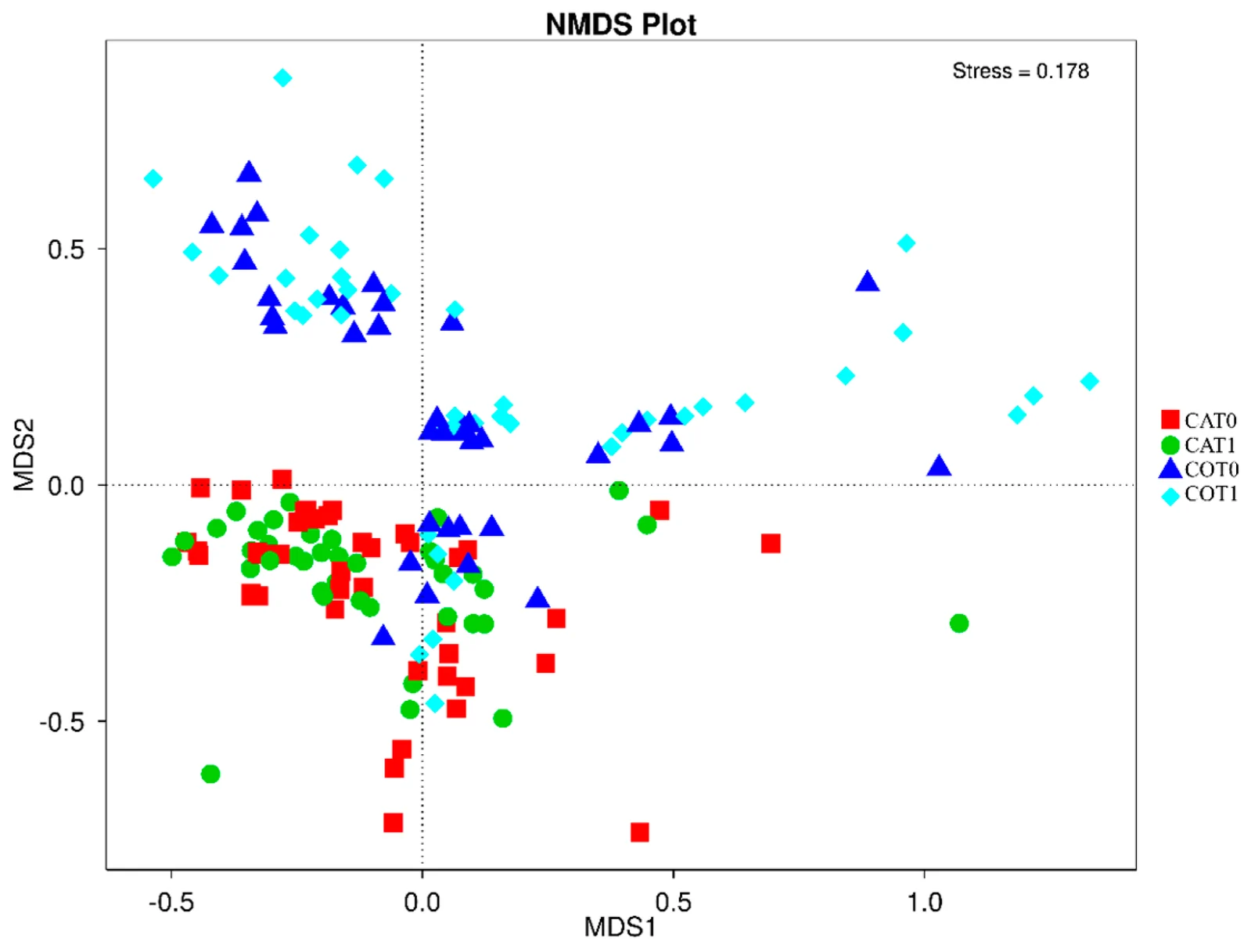
NMDS plot showing the differences in the microbiome in the different samples. The plot is based on rarefied operational taxonomic unit (OTU) abundance based on Bray–Curtis distances. Abbreviations: COT0, COT1: control non-treated patients at time 0 (0 days) and time 1 (after two weeks) from time of entrance to IRCCS Fondazione Don Carlo Gnocchi.

Moreover, the non-metric multidimensional scaling analysis (NMDS), a robust indirect gradient analysis that creates an ordination based on a dissimilarity or distance matrix [44], showed that (Figure 9) CA and CO samples clustered separately, as was also confirmed by the alpha diversity analysis. In particular, CAT0–CAT1 were close to each other and grouped in the third quadrant together with some COT0–COT1 samples; most of the COT1–COT0 samples were in the first and second quadrants.

#### 3.5.2. Eubacteria and *Lactobacillus* spp. Quantification by qPCR for the *rpo*B Gene Copy Number

The quantification of eubacteria and *Lactobacillus* spp. were expressed in *rpo*B copy number ng^−1^ DNA. In this way, the number of copies of *rpo*B represented the number of bacterial cells because the *rpo*B gene is present in only one copy per bacterial genome [45,46].

**Figure 10:**
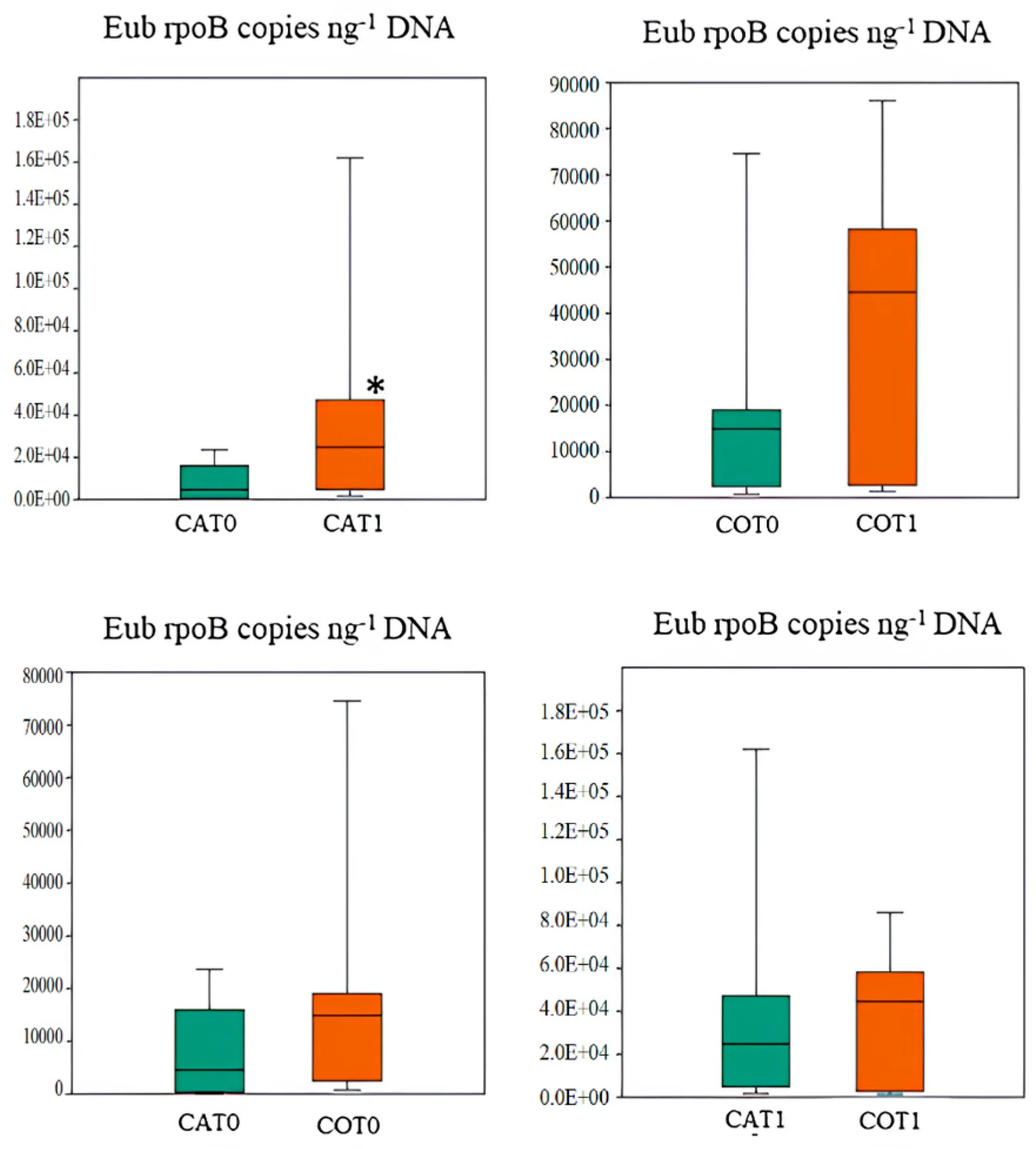
Quantification of Eubacteria by *rpo*B copy number, comparing controls (CO) and WP-treated cases (CA) at two different times (T0 and T1). Significant differences were considered for *p* < 0.05 by *t*-test analysis; * significative difference. Abbreviations: COT0, COT1: control non-treated patients at time 0 (0 days) and time 1 (after two weeks) from time of entrance to IRCCS Fondazione Don Carlo Gnocchi. CAT0, CAT1: WP-treated patients at time 0 and 1 as described above.

Based on the *t*-test analysis, there was a significant increase in the amount of CA eubacterial numbers between sampling time T0 and T1, while no significant difference in the CO group was found, as was shown in the other comparisons (Figure 10).

**Figure 11:**
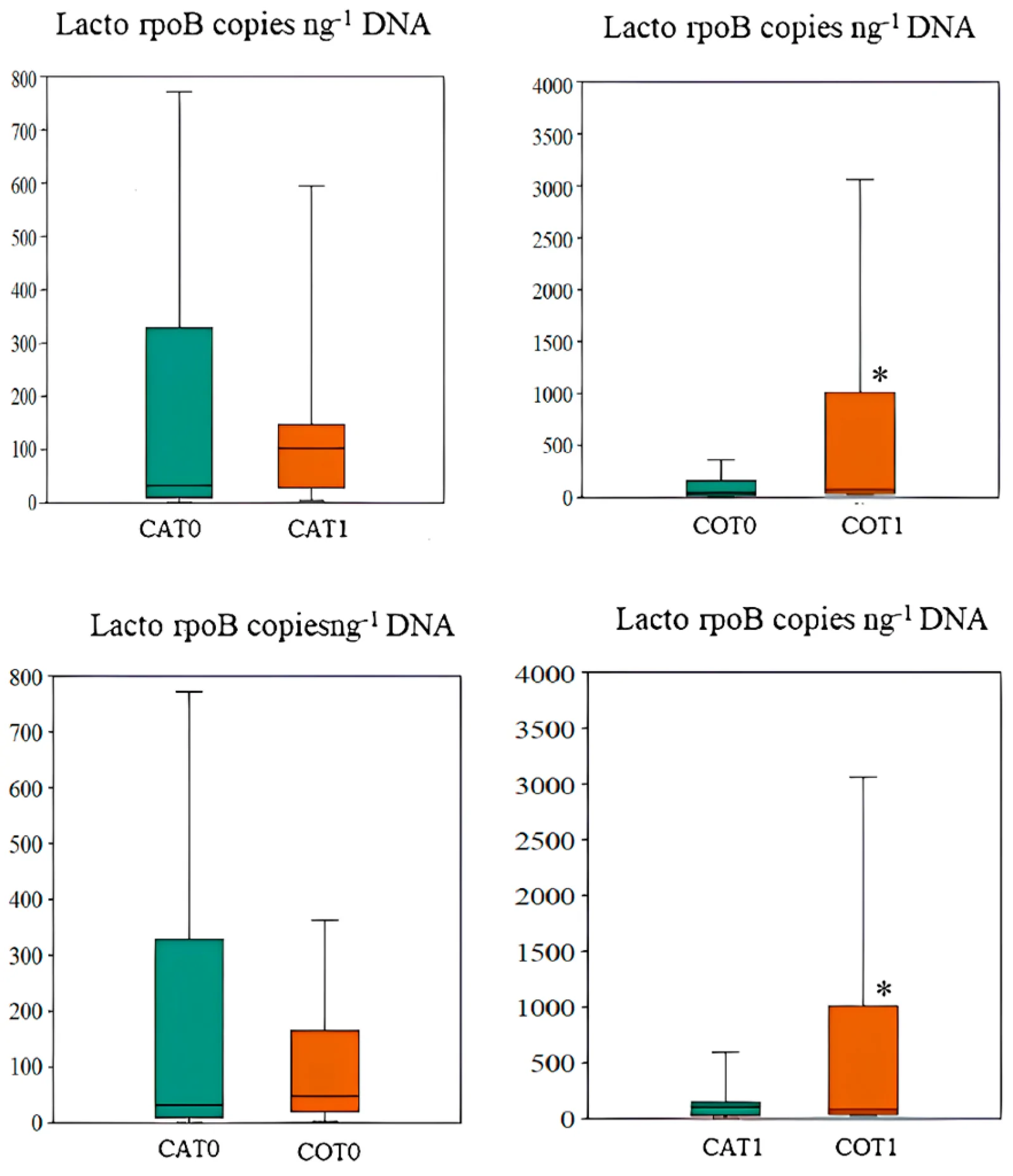
Quantification of *Lactobacillus* sp., expressed in *rpo*B copy number, comparing controls (CO) and WP-treated cases (CA) at two different times (T0 and T1). Significant differences were considered for *p* < 0.05 by *t*-test analysis; * significative difference. Abbreviations: COT0, COT1: control non-treated patients at time 0 (0 days) and time 1 (after two weeks) from time of entrance to IRCCS Fondazione Don Carlo Gnocchi. CAT0, CAT1: WP-treated patients at time 0 and 1 as described above.

The *t*-test analysis on *Lactobacillus* amount showed that there was a significant increase in the control group (COT0 and COT1) while there were no significant differences between CAT0 and CAT1, nor between CAT0 and COT0. In COT1 samples, instead, *rpo*B copies were significantly higher than CAT1 (Figure 11).

Given that the bacterial genus *Subdoligranulum* appears to play a potential role in the physiological regulation of host metabolism, we conducted—for purely exploratory purposes—a correlation analysis between this bacterial genus and the biochemical parameters IL-6, CRP, and HDL cholesterol. The results showed a trend, not significant, for these correlations (Figure S1); there is a tendency toward a negative correlation between *Subdoligranulum* and IL-6, and a tendency toward a positive correlation between *Subdoligranulum* and HDL cholesterol, although not significant.

## 4. Discussion

The exploratory results of this study suggest that oral nutritional supplementation enriched with WP, leucine, and vitamin D may modulate low-grade inflammation in older patients attending cardiac rehabilitation. Specifically, we observed a significant decrease in serum IL-6 and CRP levels in the case group over the two-week treatment period. Moreover, levels of the acute phase reactant protein ferritin decreased significantly in the cases compared to the control group. Conversely, no significant differences were observed between cases and controls regarding the pro-inflammatory cytokines IL-1β and TNF-α, or the anti-inflammatory cytokine IL-10, following two weeks of treatment.

The selective reduction in IL-6 and CRP, alongside stable TNF-α and IL-1β levels, reflects the distinct physiological kinetics of these inflammatory mediators. While IL-6 and hepatic CRP act as highly responsive acute-phase markers sensitive to short-term nutritional modulations, TNF-α and IL-1β function as primary upstream membrane-bound cytokines requiring prolonged intervention periods to manifest systemic changes. Exploratory subgroup analysis stratified by median baseline CRP (>3.0 mg/L) revealed that individuals with heightened baseline inflammatory status exhibited the greatest relative anti-inflammatory benefit from the composite supplement.

A meta-analysis carried out in 2015 found a significant decrease in serum CRP levels only when subjects received high doses of WP (>20 g/day) or presented with elevated baseline CRP levels (≥3 mg/L) [14].

Consistent with these findings, baseline mean CRP levels in our study were 3.60 mg/L in the control group and 3.79 mg/L in the case group, higher than 3 mg/L (reference values < 0.50 mg/dl). Furthermore, participants received high daily doses of WP (40 g/die).

Conversely, during a 15-day supplementation trial with 44 g of WP once daily, the authors observed a significant increase in antioxidant capacity and glutathione levels, alongside a significant increase in TNF-α levels compared to the control group, who did not receive any supplementation [47]. Similarly, a randomized clinical trial (RCT) in healthy older adults showed that IL-6, IL-1β and TNF-α levels did not change significantly following three weeks of WP isolate supplementation [2]. It is worth mentioning that both studies were conducted in healthy subjects: medical students in the former [47] and older adults in the latter [2].

Accordingly, a systematic review and meta-analysis of RCT focused on IL-6 and TNF-α across diverse populations, including older adults, postmenopausal women, overweight/obese subjects or subjects with sarcopenic obesity, patients with different conditions, such as hypertension, type 2 diabetes, or those receiving hemodialysis, reported no favorable effect on IL-6 and TNF-α following supplementation with WP not in combination with other supplements [48].

Another systematic review examining the acute/short-term effects of WP alone, or in combination with carbohydrates, on inflammatory states yielded inconclusive results [49]. However, most included studies were conducted on athletes, active adults, and young healthy males; very few studies were conducted on overweight and obese patients [49].

On the other hand, a systematic review and meta-analysis investigating the effect of intact WP supplementation on different circulating inflammatory biomarkers in older subjects reported that WP supplementation significantly lowered inflammation, depending on participant characteristics and treatment duration [15].

A significant reduction in circulating TNF-α levels was associated with a WP supplement administered to subjects aged <60 years [15]. Serum IL-6 levels were significantly decreased when WP supplements were administered to patients with sarcopenia or pre-frailty status, with a BMI ≥ 25 kg/m^2^ or with a daily dose > 30 g [15].

On the other hand, a shorter treatment duration (≤8 weeks) and a lower BMI (≤25 kg/m^2^) were associated with a significant decrease in circulating CRP levels [15].

In our study, the mean age of participants was 72 years in the control group and 69.6 years in the case group; both ages are higher when compared to participants in the Prokopidis study [15]. We speculate that a higher mean age of participants could explain no significant changes in the serum TNF-α levels of patients enrolled in our study. The WP supplement administered in our study was also combined with vitamin D3, which contributed in part to the observed reduction in inflammatory markers [11]. Previous studies investigating the effects of vitamin D3 supplementation on serum levels of inflammatory markers have yielded conflicting results [50,51].

In our study, patients in both groups received vitamin D3; however, changes in inflammatory markers levels were observed mostly in the case group, where vitamin D3 was co-administered with WP.

Beyond musculoskeletal health, extra skeletal effects of vitamin D3 have been described. Regarding the link between serum vitamin D3 levels and lipid profiles, studies reported unclear results [52]. Observational studies suggested a beneficial effect, whereas interventional studies did not observe a significant effect [52]. In our cohort, patients in the case group receiving WP and vitamin D3 exhibited distinct lipid profiles compared to the control group; specifically, HDL cholesterol increased significantly in the case group, whereas it decreased between T1 and T0 in the control group. In contrast to previous evidence [53], we observed no significant changes in total cholesterol, LDL cholesterol, and triglycerides levels between the two groups.

The intervention administered was a multi-component oral nutritional supplement containing whey protein (WP), leucine, vitamin D, fiber, and micronutrients. Consequently, the observed clinical and biomarker changes cannot be attributed solely to WP.

The molecular approach adopted—based on sequencing and qPCR-based quantification—proved sensitive enough to detect changes in the gut bacteriome in response to dietary modifications over a relatively short period (two weeks). Indeed, analysis of bacterial diversity in samples COT0 and COT1 revealed variations in bacteriome alpha diversity, a finding corroborated by the UPGMA tree based on weighted UniFrac distance. This was not accompanied by a significant increase in the abundance of eubacterial *rpo*B sequences measured by qPCR, although an upward trend was observed. In contrast, *Lactobacillus* abundance showed a significant increase between COT0 and COT1. In the WP-treated samples (CAT0 and CAT1), the change in alpha diversity was not statistically significant; however, the UPGMA tree based on weighted UniFrac distance at the phylum level showed that the CAT1 group—the only one treated with WP—diverged phylogenetically from the untreated groups, even though these patients had followed the same diet as the controls. The abundance of eubacterial *rpo*B sequences increased significantly in the CAT1 group after two weeks, whereas *Lactobacillus* abundance showed no significant change, despite a trend toward a decrease. Previous evidence on healthy obese postmenopausal women showed that after consumption of 15 g glycol-macropeptide + 10 g WP twice daily for 1 week and thrice daily for 1 week, the overall α diversity decreased [54]. Additionally, a similar decrease in α diversity was observed in overweight/obese children who received prebiotics once daily for 16 weeks [55]. A possible reason could be that specific taxa may selectively metabolize the nutrients in the WP supplement and, in a short time, increase in abundance when compared to other taxa. From these results, we can deduce that the two-week treatment with WP, added to the diet of the patients (CA), induced an increase in the gut eubacterial biomass but apparently not in *Lactobacillus* spp., in relation to the untreated patients; the CAT1 eubacterial biomass was phylogenetically and compositionally different from that of the untreated patients. WP supplementation seems to mask diet-induced microbial changes; conversely, untreated samples exhibited significant changes in bacterial composition over both the short (2 weeks) and long term [23]. In animal models, whey protein supplementation frequently results in increased gut microbiota diversity, increased growth of beneficial microbial species and decreased markers of inflammation. Thus, they can promote the growth of beneficial organisms and can be considered as potential prebiotic dietary supplements. On the other hand, some studies on overweight or obese adult humans found that whey protein did not significantly alter the gut microbiome [21]. The discussion remains open concerning CAT0 and COT0 at time zero, upon entry, which showed a significantly different composition. We would have expected, instead, a similarity in alpha diversity. This evidence could be due to the large degree of variation in human gut microbiomes, in human eating behaviors, environmental factors, and genetics [19]. Considering the way patients were selected, we do attribute this result to randomness, but suggestions are welcome. With regard to sequencing results, we focused attention on the genus *Subdoligranulum* and the biochemical parameters IL-6, PCR, and HDL cholesterol. In fact, this bacterial genus may have a potential role in the physiological regulation of host metabolism. For example, it can produce short-chain fatty acids that serve as energy sources for the intestinal epithelium, can reduce inflammation but increase intestinal motility [56]. Our results showed a tendency to a negative correlation between *Subdoligranulum* and IL-6, and a positive correlation between *Subdoligranulum* and HDL cholesterol, although not significant. But it is true that our observations cover a relatively short period (two weeks), which may not have shown any significant change. However, there are still not many studies on the behavior of *Subdoligranulum* in the human gut, while those in mice are slightly more numerous. However, although our data are not statistically significant, they have been shown to be in line with the most recent results showing that *Subdoligranulum* correlated negatively with IL-6 while correlating positively with HDL cholesterol in humans and in mice [56]. In any case, it might be interesting to further study the presence and activity of this bacterial genus in the human intestine, since it has been associated with the control of obesity and, in general, with good metabolic health in human adults [57,58,59].

It should be noted that this study has some limitations, largely related to the sample size (*N* = 27), which characterizes this study as a preliminary/exploratory pilot trial; caution must be exercised to avoid over-extrapolating these findings to broader populations.

Additionally, the discrepancy between the distribution of men and women between the two groups—with the control group having more female patients (54%) compared to the case group (21%)—is another limitation. It is well known that premenopausal status protects women from cardiovascular diseases, having female sex hormone-related antioxidant, anti-inflammatory, antiproliferative, and dilating effects [60]. Conversely, after menopause, the protective effect of female sex hormones is lost [60]. After menopause, women may show diverse pro-inflammatory cytokines, and, as a rule, develop later and more severe cardiovascular diseases with worse outcomes [61]. However, the small size of our sample limits further subgroup, sex-stratified statistical analyses. It could be interesting to strengthen our preliminary results in a future, larger, randomized sex-stratified clinical trial.

Notably, in our study, adherence to supplement prescription was reported by the nurse staff and not evaluated through dietary assessment.

Additionally, the intervention administered was a multi-component oral nutritional supplement; consequently, the observed clinical and biomarker changes cannot be attributed solely to WP.

Another limitation of our study was the lack of long-term follow-up data. Indeed, it would be interesting to plan a larger study including a longer intervention period and a long-term follow-up.

Conversely, a major strength of this study is the scarcity of human clinical data regarding the concurrent effects of WP on inflammation, cardiometabolic health, and gut microbiota.

Additionally, all enrolled patients received a standardized hospital diet with comparable macro- and micronutrient composition, as well as a standardized exercise-based physiotherapy program comparable in terms of session duration and weekly frequency.

Finally, the open-label design introduces an inherent risk of performance and detection bias. Participant and clinical staff awareness of group allocation could have subtly influenced unmeasured lifestyle factors such as spontaneous physical activity, secondary dietary choices, or psychological motivation during rehabilitation exercises. Although standardized rehabilitation protocols and objective automated laboratory assays were utilized to minimize detection bias, future studies should employ double-blind placebo-controlled designs to eliminate these behavioral confounders.

## 5. Conclusions

In older adults with cardiovascular disease, chronic low-grade systemic inflammation (inflammaging) is intricately linked with gut microbiota dysbiosis. Reduced cardiac index and systemic endothelial dysfunction often lead to intestinal hypoperfusion and altered mucosal integrity. This barrier breakdown facilitates the translocation of gut-derived endotoxins (e.g., lipopolysaccharides) into circulation, fueling systemic inflammatory cascades (elevated IL-6, CRP) that exacerbate sarcopenia and delay physical recovery. Although cardiac rehabilitation programs effectively improve functional capacity, exercise alone may not fully attenuate persistent metabolic and microbial imbalances. Combining structured exercise with targeted nutritional supplementation represents a promising therapeutic avenue to restore intestinal homeostasis and mitigate systemic immune activation in this vulnerable population.

The observed variations in gut bacteriome composition suggest a potential susceptibility of the microbiota to short-term modifications; however, they do not establish a direct causal link or confirm their clinical relevance. Larger-scale, controlled clinical trials are required to validate these observations.

In polymorbid patients receiving polypharmacy during rehabilitation, non-pharmacological dietary interventions devoid of side effects may represent a crucial strategy to reduce risk factors, shorten lengths of hospital stay, alleviate comorbidity burden, and reduce healthcare costs for national health systems.

## Figures and Tables

**Table 1:** Baseline demographics and clinical characteristics of patients.

Characteristics	Control Group (n = 13)	Case Group (n = 14)
Age, years	72 ± 8.7 (range 55–84)	69.6 ± 7.5 (range 55–77)
Gender, male/female (%)	M 6 (46%)/F 7 (54%)	M 11 (79%)/F 3 (21%)
Dyslipidemia, n (%)	7 (54%)	5 (36%)
Angina Pectoris, n (%)	/	/
Valvular Disease, n (%)	11 (85%)	13 (93%)
Miocardial Infarction, n (%)	1 (8%)	1 (7%)
Heart Failure, n (%)	4 (31%)	/
Arterial Hypertension, n (%)	10 (77%)	7 (50%)
Type 2 Diabetes *, n (%)	3 (23%)	1 (8%)
Smokers + Ex-Smokers, n (%)	6 (46%)	7 (50%)

* Diagnosis of type 2 diabetes: fasting plasma glucose > 126 mg/dl; according to Associazione Medici Diabetologi (AMD)-Società Italiana di Diabetologia (SID) Italian Society of Diabetology [41]; diagnosis of arterial hypertension was defined as systolic blood pressure of ≥140 mmHg or diastolic blood pressure of ≥90 mmHg, according to the task force on the management of elevated blood pressure and hypertension of the European Society of Cardiology (ESC) and endorsed by the European Society of Endocrinology (ESE) and the European Stroke Organisation (ESO) [42]. Values are mean ± SD.

## Data Availability

The authors ensure that the sharing of data was carried out in accordance with the consent provided by participants on the use of confidential data. They also ensure that the publication of such data does not compromise the anonymity of the participants or breach local data protection laws.

## Supplementary Materials

The following supporting information can be downloaded at: https://www.mdpi.com/article/10.3390/biom16101484/s1, Figure S1: Correlation between Subdoligranulum and biochemical parameters.

## Abbreviations

The following abbreviations are used in this manuscript:

ANOVA	Analysis of variance
BMI	Body Mass Index
CA	Case Group
CAT0	Case Group at time 0
CAT1	Case Group at time 1
CO	Control Group
COT0	Control Group at time 0
COT1	Control Group at time 1
CRP	C-Reactive Protein
Ct	Threshold Cycle
CVD	Cardiovascular Disease
DCNS	Dietetic and Clinical Nutrition Service
DNA	Deoxyribonucleic Acid
ETS	Ente Terzo Settore–Third Sector Entity
FDG	Fondazione Don Carlo Gnocchi-
IL-1β	Interleukin-1β
IL-6	Interleukin-6
IL-10	Interleukin-10
IRCCS	Istituto di Ricovero e Cura a Carattere Scientifico–Scientific Institute for Research, Hospitalization and Care
LAB	*Lactic* Acid Bacteria
NCDs	Non-Communicable Diseases
NMDS	Non-Metric Multidimensional Scaling Analysis
OTU	Operational Taxonomic Unit
PD	Phylogenetic Diversity
qPCR	Quantitative-Polymerase Chain Reaction
RCT	Randomized Clinical Trial
SEM	Standard Error of the Mean
spp.	Species
T0	Time 0
T1	Time 1
TNF-α	Tumor Necrosis Factor-α
UPGMA	Unweighted Pair-Group Method with Arithmetic Mean
WP	Whey Proteins
